# No Significant Changes in Addictive and Problematic Behaviors During the COVID-19 Pandemic and Related Lockdowns: A Three-Wave Longitudinal Study

**DOI:** 10.3389/fpsyg.2022.837315

**Published:** 2022-04-13

**Authors:** Mónika Koós, Zsolt Demetrovics, Mark D. Griffiths, Beáta Bőthe

**Affiliations:** ^1^Doctoral School of Psychology, Eötvös Loránd University, Budapest, Hungary; ^2^Institute of Psychology, Eötvös Loránd University (ELTE), Budapest, Hungary; ^3^Centre of Excellence in Responsible Gaming, University of Gibraltar, Gibraltar, Gibraltar; ^4^International Gaming Research Unit, Psychology Department, Nottingham Trent University, Nottingham, United Kingdom; ^5^Département de Psychologie, Université de Montréal, Montreal, QC, Canada

**Keywords:** addictive behaviors, compulsive sexual behavior disorder, COVID-19, gambling disorder, Internet gaming disorder, longitudinal, problematic pornography use, problematic social media use

## Abstract

**Introduction:**

The COVID-19 outbreak and related lockdowns brought substantial changes in people’s lives and led to concerns about possible increases of addictive behaviors at the initial stages of the pandemic. To examine these concerns, the aim of the present study was to assess longitudinal changes in addictive and problematic behaviors (i.e., problematic social media use, Internet gaming disorder, gambling disorder, problematic pornography use, and compulsive sexual behavior disorder) over time during the COVID-19 pandemic.

**Methods:**

Three waves of data collection took place in different stages of the COVID-19 outbreak in Hungary in a general population, from the first wave of lockdowns to the second and third waves of restrictions (May, 2020; N_*T*1_ = 1747; June–August, 2020; N_*T*2_ = 656; January, 2021; N_*T*3_ = 411). Latent growth curve models were calculated to assess the potential changes in addictive and problematic behaviors over time.

**Results:**

Latent growth curve models showed that the sample varied in their initial scores, but there were no significant changes over time in any of the examined behaviors, except for compulsive sexual behavior disorder, which demonstrated a small but significant increase (i.e., positive and significant slope factor). However, the rate of this change was negligible. Overall, there were no noteworthy changes over time regarding any of the examined addictive and problematic behaviors.

**Conclusion:**

Contrary to initial concerns, no substantial changes over time were observed regarding the examined addictive behaviors during the COVID-19 pandemic and related lockdowns. These findings indicate that those who had no previous problem with these addictive behaviors, might have not developed a problem, and those who had problem with either of the behaviors previously, might have not experienced a significant increase in their symptoms.

## Introduction

The spread of the COVID-19 virus and the restrictions that followed substantially changed everyday life worldwide, and individuals had to adapt to these changes quickly. Nationwide lockdowns were enforced, and the major areas of people’s lives were moved to the online sphere. The new measures such as physical distancing, lockdowns, quarantining, and working and/or learning from home, led to the deprivation of many basic psychological needs (e.g., being able to physically connect to others, being intimate, the subjective feeling of freedom, and autonomy) (Ryan and Deci, 2017). Consequently, the question has arisen of how these changes and new stressors might have affected individuals’ mental health (Armbruster and Klotzbücher, 2020). Previous studies suggest that isolation might have severe negative effects on mental health, such as elevated stress or anxiety levels (Bai et al., 2004; Wu et al., 2009; Liu et al., 2012; Sprang and Silman, 2013; Brooks et al., 2020; Purssell et al., 2020; Henssler et al., 2021). Furthermore, there is already a great body of evidence on the negative impacts of the COVID-19 pandemic on mental health and general well-being (Chen L. et al., 2021; Daly et al., 2021; Kira et al., 2021; Magson et al., 2021; O’Connor et al., 2021; Ravens-Sieberer et al., 2021; Wang et al., 2021). In addition to lockdown-related isolation, several other stressors could be present during these times, including health anxiety, grief, and financial problems (Kira et al., 2021).

Individuals with already existing mental health problems are considered one of the main risk groups affected by isolation (Burton et al., 2021; Gillard et al., 2021), as in some other countries, in-person mental health services have also become limited in Hungary during the pandemic, making relapse prevention even more challenging (Columb et al., 2020; Kar et al., 2020; Liese and Monley, 2021). Moreover, those living with addictive disorders might be at an even higher risk of experiencing decline of their mental health or relapsing (Bonny-Noach and Gold, 2021), since leisure activities are limited to at-home activities (Kar et al., 2020), creating an environment, where avoiding cue stimuli of the given problematic behavior is extremely difficult. Following from learning theories (where addiction involves learning associations between cues, responses and reinforcements), limiting the exposure to these cues and reinforcing new, competing behaviors (which could serve as a healthy alternative) (Hyman et al., 2006; West, 2013) would be key in relapse prevention, although in lockdown situations, these activities appear to be difficult to pursue. However, the imitation theories of addiction, and more specifically, social learning theory (which describes the beginning of an addiction in the context of imitating behavioral patterns, with assimilation of identities) (Heyes, 2011; Smith, 2021) might not support this notion, since the possibilities of observing and mimicking others’ behavior is limited during social distancing. From another perspective, based on the reflective choice theories of addiction, examining (actual or perceived) cost and benefits of the decisions and behaviors (Lussier et al., 2006; Davies, 2013) might be biased if the circumstances are psychologically challenging, such as during a worldwide pandemic. It might be easier to underestimate the risks and the negative consequences of the given behavior (e.g., impairment in social relations and hobbies), resulting in possible cognitive biases (Field and Cox, 2008; Olusanya, 2014).

In association with lockdowns, a significant proportion of life shifted to online platforms (Király et al., 2020). Although the use of the information and communication technology (ICT) is inevitable and provides a great solution to the current unprecedented situation (e.g., working or being taught from home), increased screen-based activities might be a risk factor for developing problematic use, or cause a relapse cycle for those who were already involved in potentially addictive behaviors (Fineberg et al., 2018; King et al., 2020; Ko and Yen, 2020; Mestre-Bach et al., 2020; Singh et al., 2020; Masaeli and Farhadi, 2021). Although more frequent use of ICT is not a sufficient criterion for defining problems with these behaviors, using them to cope with the elevated stress levels and to avoid adverse psychological states and moods are among the strongest motivations underlying problematic and addictive behaviors (Sinha, 2001; Király et al., 2015; Bőthe et al., 2021a,^2022^). This phenomenon might be explained by the emotional self-medication theory (Khantzian, 1997; Kor et al., 2013; Torres and Papini, 2016). This theory proposes that using a given activity to reduce stress and avoid negative emotions may contribute to the severity of the addictive behavior, by maintaining that behavior via negative reinforcement (Blume et al., 2000). Moreover, as in some other countries, in-person mental health services have become limited in Hungary during the pandemic, making relapse prevention even more challenging (Columb et al., 2020; Kar et al., 2020; Liese and Monley, 2021).

At the initial stages of the pandemic, it was proposed in the literature that not just the frequency of specific addictive behaviors, but also the severity of problematic behaviors might increase during the COVID-19 pandemic and related lockdowns due to the aforementioned processes (Király et al., 2020; Mestre-Bach et al., 2020; Singh et al., 2020; Awan et al., 2021). However, the methodologies and the research designs of most studies that tried to provide empirical answers to these propositions varied. In the case of some screen-based activities, studies have analyzed the user trends and behavioral patterns. More specifically, pornography searches showed a clear peak during lockdowns (Pornhub Insights, 2020; Zattoni et al., 2021), while gambling (e.g., online betting and online casino use) showed no changes over time, or even decreased (Auer et al., 2020; Lindner et al., 2020; Auer and Griffiths, 2021).

There is a large body of cross-sectional survey studies conducted during the pandemic, working with retrospective methods (i.e., asking participants to think back to the pre-pandemic times and comparing it to current use) regarding the frequency of the given behavior (e.g., Håkansson, 2020; Gainsbury et al., 2021; Lugo et al., 2021; Sallie et al., 2021; Wardle et al., 2021). However, there is a relative lack of longitudinal studies. Teng et al. (2021) examined 903 adolescents regarding Internet gaming disorder. They found significant increases both in gaming behavior frequency and severity of gaming disorder over a period of 6 months (conducting data collections before and after the COVID-19 outbreak), which was predicted by depressive and anxiety symptoms, but not inversely. These findings could be interpreted as adolescents used gaming to cope with negative feelings and emotions during these stressful times, resulting in more severe symptoms of gaming disorder. This is in line with previous studies indicating that coping and escapism motivations can be strong indicators of problematic Internet gaming (Király et al., 2015; Schneider et al., 2018). In contrast, Chen I.-H. et al. (2021) found that Chinese students reported decreased problematic social media use and problematic gaming during the COVID-19 outbreak period, compared to pre-pandemic baseline measures.

Regarding problematic pornography use, Grubbs et al. (2021) collected data from a nationally representative sample of US adults before and during the COVID-19 pandemic over a period of 15 months, and reported a downward trend in pornography use frequency over time (Grubbs et al., 2021). Although there was an initial peak of use during the first lockdown (April and May 2020), even for those participants who reported a self-perceived increase in their use, pornography use frequency returned to the same level after 6 months. Problematic pornography use did not change in the case of women, and decreased over time in the case of men between August of 2019 and October of 2020 (Grubbs et al., 2021). Another repeated-measure study of adolescents’ pornography use that compared pre-pandemic and during pandemic use had similar findings (Bőthe et al., 2022). Namely, neither the frequency of use, motivations, nor problematic use changed over time.

No longitudinal study has been published examining other problematic or addictive behaviors, such as problematic social media use, gambling disorder, or compulsive sexual behavior disorder symptoms during the COVID-19 pandemic. However, relying solely on cross-sectional and retrospective data might lead to biased conclusions, since causality cannot be inferred from cross-sectional research designs (Rindfleisch et al., 2008). Moreover, based on previous findings, intense and time-consuming behaviors might not be inherently problematic or addictive and therefore the frequency or quantity of engagement in a given behavior should be considered as a peripherical symptom of problematic use (Charlton and Danforth, 2007; Billieux et al., 2019; Bőthe et al., 2020a). Consequently, examining the aforementioned behaviors without assuming that an elevated frequency of use would automatically lead to problematic use (Bőthe et al., 2020c) might contribute to the scientific discussion of the possible impacts of the pandemic. The present study aims to fill this gap by monitoring longitudinal changes in addictive and problematic behaviors (i.e., problematic social media use, Internet gaming disorder, and gambling disorder) over time during the COVID-19 pandemic to better understand the potential impact of the stay-at-home policies and related elevated stress levels. As no prior longitudinal reports are available about these problematic behaviors during the COVID-19 pandemic, the present study was conducted in an exploratory manner.

## Materials and Methods

### Procedure and Participants

Online data were collected via a popular Hungarian news website. The first data collection wave was conducted in May 2020, immediately after the Hungarian government declared a state of emergency. Schools, universities, and leisure establishments were closed, individuals were advised to stay at home (except for essential workers) and practice physical distancing. The second data collection wave was during the summer of 2020 (July–August), when almost all of these restrictions were lifted. Although schools were not open due to the summer vacation period, cinemas, pubs, and restaurants reopened, and the state of emergency was withdrawn. The third wave of the data collection was conducted in January 2021, when new restrictions were introduced on top of the already existing ones from the spring of 2020. For example, individuals could not leave their homes between 8 p.m. to 5 a.m. (i.e., a curfew was introduced).

Participants were informed about the aims of the study and informed consent was obtained. The survey took approximately 30 min to complete and only adults (18 years old and above) were invited to participate. Participants were asked to provide their email addresses if they agreed to participate in the next data collection waves, but it was not obligatory. They were assured that their personal information (i.e., email address) was handled according to the General Data Protection Regulation (GDPR) and stored in a different dataset than their answers to the surveys, guaranteeing their anonymity. A unique and reproducible identification code (e.g., a letter from their mother’s name, a number from their year of birth) was collected to match their answers in the different data collection waves. The study was approved by the Institutional Review Board of the research team’s university and conducted in accordance with the Declaration of Helsinki.

A total of 1,747 participants completed the survey at the first data collection wave. Seven participants were excluded for being underaged. A total of 1,091 individuals agreed to participate in the second wave of data collection (i.e., provided their email address during the first data collection wave and agreed to be contacted for follow-up survey), of which 656 filled out the survey during the second data collection wave, and 411 also completed the survey at the third data collection point. The gender ratio was equal at the first data collection wave (*N*_*males*_ = 882; 50.5%) and the mean age of the sample was 41.96 years (SD = 12.52). Detailed sociodemographic characteristics of the participants are presented in Supplementary Appendix A.

### Measures

Before each psychometric scale assessing a problematic behavior, a screening question was presented whether the individual had engaged in a given behavior in the past 12 months (“*Did you __ in the last 12 months?*”). If participants engaged in the specified behavior in the past 12 months, they then completed a psychometric scale assessing problematic or addictive engagement in that particular behavior in the past 7 days. The reliability of all measures was acceptable (ɑ = 0.55 to 0.90; see Table 1). The following measures were used to assess the five problematic behaviors:

**TABLE 1:** Descriptive statistics, normality indices, and psychometric properties of the scales.

		Problematic social media use (BSMAS)	Online gaming disorder (IGDT-10)	Gambling disorder (PGSI)	Problematic pornography use (PPCS-6)	Compulsive sexual behavior disorder (CSBD-19)
*N*	T1	1,188	680	547	712	835
	T2	534	219	236	278	408
	T3	340	144	166	182	235
*M* (SD)	T1	9.61 (3.98)	0.36 (0.96)	9.27 (1.60)	10.02 (5.75)	24.31 (6.75)
	T2	9.13 (3.45)	0.25 (0.84)	9.16 (0.77)	9.87 (5.56)	23.39 (6.25)
	T3	9.58 (3.77)	0.27 (0.93)	9.12 (0.49)	9.81 (5.66)	22.43 (5.84)
Observed range	T1	6–28	0–9	9–27	6– 42	19–66
	T2	6–26	0–8	9–17	6–40	19–55
	T3	6–24	0–7	9–13	6–40	19–62
Cronbach’s alpha	T1	0.80	0.71	0.93	0.85	0.90
	T2	0.77	0.75	0.71	0.84	0.90
	T3	0.80	0.78	0.55	0.86	0.90
Composite reliability index (CRI)	T1	0.81	0.76	0.91	0.86	0.90
	T2	0.78	0.79	0.73	0.86	0.91
	T3	0.81	0.84	NA	0.88	0.92
Skewness (SE)	T1	1.30 (0.07)	4.02 (0.09)	8.16 (0.10)	1.97 (0.09)	2.06 (0.09)
	T2	1.36 (0.11)	5.46 (0.16)	7.28 (0.16)	2.18 (0.15)	2.20 (0.12)
	T3	1.182 (0.13)	5.07 (0.20)	4.98 (0.19)	2.19 (0.18)	3.16 (0.16)
Kurtosis (SE)	T1	1.36 (0.14)	20.92 (0.19)	75.29 (0.21)	4.23 (0.18)	5.17 (0.17)
	T2	1.76 (0.21)	38.69 (0.33)	61.65 (0.32)	5.63 (0.29)	5.43 (0.24)
	T3	0.93 (0.26)	29.93 (0.40)	4.98 (0.19)	5.67 (0.36)	13.20 (0.32)

*BSMAS, Bergen Social Media Addiction Scale; IGDT-10, Internet Gaming Disorder Test-10; PGSI, Problem Gambling Severity Index; PPCS-6, Problematic Pornography Consumption Scale – 6; CSBD-19, Compulsive Sexual Behavioral Disorder Scale; M, mean; SD, standard deviation; SE, standard error.*

*Bergen Social Media Addiction Scale* (BSMAS; Andreassen et al., 2017; Hungarian version: Bányai et al., 2017). The six-item BSMAS was used to assess problematic social media use regarding several platforms (e.g., Facebook and Twitter). The items (e.g., “*How often have you become restless or troubled if you have been prohibited from using social media?*”) are based on the component model of addictions (Griffiths, 2005). Participants indicate their answers on a five-point scale (1 = “very rarely”; 5 = “very often”). Scores are summed, and higher scores indicate higher degree of problematic social media use.

*Internet Gaming Disorder Test-10* (IGDT-10; Király et al., 2019). The ten-item IGDT-10 was used to assess problematic gaming. The ten items cover the nine diagnostic criteria of Internet gaming disorder in the DSM-5 (American Psychiatric Association, 2013) by combining items 9 and 10 during the scoring, since they assess the same criterion (e.g., “*Have you risked or lost a significant relationship because of gaming?*”). Although participants indicate the frequency of the given statements on a three-point scale (0 = “never”; 1 = “sometimes”; 2 = “often”), items were recoded as dichotomous variables (1 = “yes’; 0 = “no”) to resemble the structure of the DSM-5 criteria for further analyses, in line with the recommendations of the original validation paper (Király et al., 2017). Scores are summed, and higher scores indicate a higher degree of problematic gaming.

*Problem Gambling Severity Index* (PGSI; Holtgraves, 2009; Hungarian version: Gyollai et al., 2013). The nine-item PGSI was used to assess gambling disorder. Four items assess gambling behavior (e.g., “*How often have you go back another day to try to win back the money you lost?*”), and five items assess related negative consequences (e.g., “*How often has your gambling caused any financial problems for you or your household?*”). Participants indicate their answers on a four-point scale (0 = “*never*”; 3 = “*almost always*”). Scores are summed, and higher scores indicate a higher degree of problematic gambling.

*Problematic Pornography Consumption Scale* (PPCS-6; Bőthe et al., 2021b). The six-item PPCS-6 was used to assess problematic pornography use. The items (e.g., “*When I vowed not to watch porn anymore, I could only do it for a short period of time*”) are based on the component model of addictions (Griffiths, 2005). Participants indicate their answers on a seven-point scale (1 = “never”; 7 = “very often). Scores are summed, and higher scores indicate higher levels of problematic pornography use.

*Compulsive Sexual Behavior Disorder Scale* (CSBD-19; Bőthe et al., 2020b). The 19-item CSBD was used to assess compulsive sexual behavior. The items are based on those in the ICD-11 (11th Revision of the International Classification of Diseases; World Health Organization, 2019b) and assesses five factors (control, salience, relapse, dissatisfaction, and negative consequences). The items (e.g., “*I could not control my sexual cravings and desires*”) are answered on a four-point scale (1 = “totally disagree”; 4 = “totally agree”). Scores are summed, and higher scores indicate higher levels of compulsive sexual behavior.

### Statistical Analyses

SPSS 28 (SPSS Inc., Chicago, IL, United States) was used for cleaning and organizing data, and for descriptive statistics, normality indices (i.e., skewness and kurtosis values), attrition analysis, and correlations. In the attrition analysis, three groups of participants (participants who completed the survey, those who dropped-out at T2, and those who dropped-out at T3) were compared regarding their demographic characteristics (e.g., gender, age, relationship status, and sexual orientation), using one-way ANOVAs (or non-parametric test if the assumptions were not met) with *post hoc* tests for continuous variables, and χ^2^ tests for categorical variables. All other analyses were conducted using Mplus 8 (Muthén and Muthén, 1998-2018).

First, confirmatory factor analyses were conducted to obtain factor scores for all constructs, as factor scores have the advantage of providing some control for measurement error by allocating more weight to the items with lower error variances, compared to manifest scale scores (Skrondal and Laake, 2001). These factor scores were used for a series of latent growth curve (LGC) analyses to examine the change over time in the aforementioned potentially addictive behaviors, with the assumption of linear growth trajectories. For confirmatory factor analyses, the Weighted Least Squares Means and Variance adjusted estimator (WLSMV) was used. Goodness of fit were determined by commonly used indices (Brown, 2015; Kline, 2015): the χ^2^ values, the Root-Mean-Square Error of Approximation (RMSEA; ≤0.06 for good, ≤0.08 for acceptable), the Tucker-Lewis Index (TLI; ≥0.95 for good, ≥0.90 for acceptable) and the Comparative Fit Index (CFI; ≥0.95 for good, ≥0.90 for acceptable) with 90% confidence intervals. However, when evaluating RMSEA values, a number of things need to be taken into account including the sample size and degrees of freedom. Moreover, the model specification of the given model, and multiple fit indices should be examined simultaneously when deciding about a given model’s adequacy (Chen et al., 2008; Kenny et al., 2015). LGC models were used to examine the changes over time in problematic social media use, Internet gaming disorder, gambling disorder, problematic pornography use, and compulsive sexual behavior disorder.

The Robust Maximum Likelihood estimator (MLR) was used for the curve models to account for the natural non-normality of the examined constructs. To determine goodness-of-fit, the same indices were taken into consideration as in case of the confirmatory factor analyses. The time differences between data collection waves were accounted for in the analyses, linear slopes were defined as a baseline measurement (T1) fixed to two (given that the first data collection wave started 2 months after the start of the pandemic), the second measurement point (T2) was fixed to four (i.e., the second data collection wave took place 4 months after the start of the pandemic), while the third measurement point (T3) was fixed to nine (i.e., the third data collection wave took place 9 months after the start of the pandemic), representing the months that passed between them.

Additional LGC models were specified for each construct, where the linear slope factors of the second data collection time (T2) were freely estimated, considering the lifted restrictions during that time. In these alternative models, the possibility that data could deviate from the linear trajectory at T2 was allowed (Grubbs et al., 2021). To compare the nested models, χ^2^ difference testing was performed (Bryant and Satorra, 2012). Considering the high drop-out rates (ranging from 52.2 to 68.8% at T2, and from 29.7 to 42.4% at T3), the Full Information Maximum Likelihood method (Newman, 2003; Newman, 2014; Lang and Little, 2018) was used to handle missing data.

## Results

### Descriptive Statistics and Correlations

Attrition analysis was conducted regarding participants demographic characteristics (e.g., gender, age, and sexual orientation) and levels of problematic and addictive behaviors at the baseline data collection. Three groups of participants were compared: those who completed the follow-up survey, those who dropped-out at the second data collection, and those who dropped-out at the third. Since the assumptions for one-way ANOVAs were not met in any case, non-parametric tests were used (independent samples Kruskal–Wallis test) for continuous variables and χ^2^ tests were used in case of categorical variables. Only gender and the initial scores of problematic social media use showed significant differences between participants who dropped out from the study and those who completed the follow-ups, but the effect sizes remained small in both cases, indicating negligible differences. Participants who stayed in the study reported higher scores of problematic social media use, and men were more likely to drop out of the study. The detailed results are presented in Supplementary Appendix B.

**FIGURE 1:**
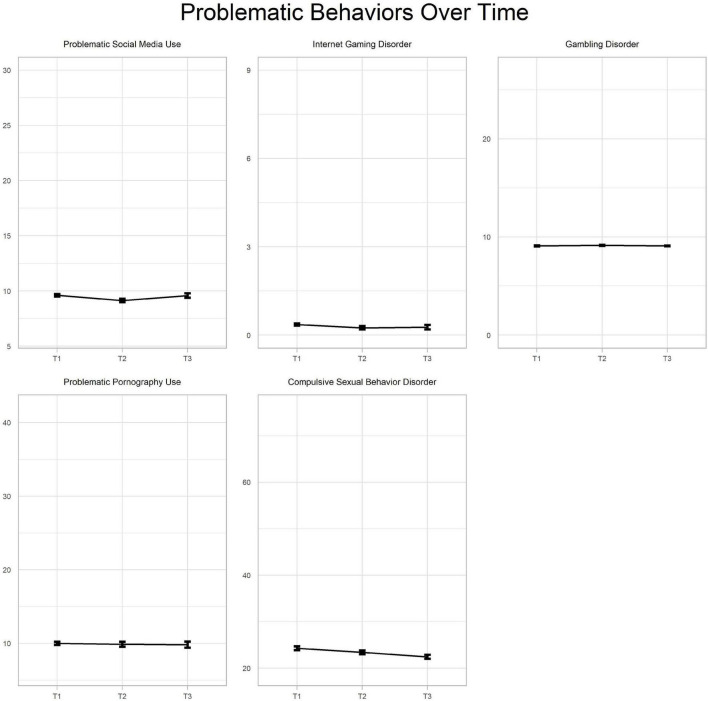
Visual representation of the problematic behaviors over time. X-axis values should be interpreted as follows: T1 = May 2020, T2 = July–August 2020, T3 = January 2021.

Descriptive data and correlations between the measured constructs at T1, T2, and T3 are presented in Tables 1, 2. The mean scores of the given problematic behaviors in the three time points are presented in Figure 1. Due to high floor effects, Spearman’s Rhos were calculated. In general, positive and moderate-to-strong associations were observed between the majority of the examined variables. However, in the case of the third data collection wave, higher numbers of associations were non-significant due to the smaller sample sizes. Sexuality-related behaviors had positive and strong associations at all data collection waves. Internet gaming disorder had positive and moderate-to-strong associations with every other construct in both T1 and T2 data collections. These associations were higher in the case of gambling disorder and problematic pornography use than with compulsive sexual behavior disorder and problematic social media use. Problematic social media use had the weakest associations with all other variables, ranging from 0.11 to 0.30.

**TABLE 2:** Correlations between the scales in the three data collection waves.

	Problematic social media use	Online gaming disorder	Gambling disorder	Problematic pornography use
Online gaming disorder	0.26**/0.17*/0.23*	–		
Gambling disorder	0.12*/0.04/0.00	0.38**/0.76**/−0.06	–	
Problematic social media use	0.14**/0.30**/0.29**	0.39**/0.52**/0.10	0.40**/0.25**/0.08	–
Compulsive sexual behavior disorder	0.11**/0.27**/0.17**	0.36**/0.26**/0.02	0.43**/0.25**/−0.02	0.54**/0.57**/0.76**

The three values in each box are the correlations at the three data collections (i.e., T1/T2/T3).*p < 0.05, **p < 0.01.

### Growth Curve Models

The goodness of fit indices for all confirmatory factor analyses and LGC models are presented in Tables 3, 4. The models consistently showed acceptable fit to the data. In case of every problematic behavior examined, two separate LGCMs were specified: one in which all time-points were fixed to a linear growth trend, and an alternative model, allowing the second time-point (T2) to vary freely, due to the differences in the lockdown-related restrictions at that time-point. Chi-square difference tests of model fit^1^ showed that the alternative model fits were not significantly better than the originals’ fit. Therefore, the fixed models were retained for each behavior. All models’ goodness-of-fit indices are presented in Table 3.

**TABLE 3:** Goodness-of-fit statistics for the estimated models.

Model	χ^2^	df	CFI	TLI	RMSEA	90% CI of RMSEA
Problematic social media use CFA, T1	70.154**	9	0.986	0.976	0.076	0.060–0.093
Problematic social media use CFA, T2	73.267**	9	0.957	0.928	0.116	0.092–0.141
Problematic social media use CFA, T3	37.659**	9	0.979	0.965	0.097	0.066–0.130
Problematic social media use LGC	15.725**	1	0.959	0.876	0.108	0.065–0.158
Problematic social media use LGC alternative model	54.844**	0	0.846	1.000	0.000	0.000–0.000
Online gaming disorder CFA, T1	40.611*	27	0.989	0.985	0.027	0.004–0.043
Online gaming disorder CFA, T2	25.796	27	0.989	0.985	0.027	0.004–0.043
Online gaming disorder CFA, T3	43.901*	27	0.984	0.978	0.066	0.026–0.100
Online gaming disorder LGC	2.550	1	0.965	0.895	0.046	0.000–0.119
Online gaming disorder LGC alternative model	0.000**	0	1.000	1.000	0.000	0.000–0.000
Gambling disorder CFA, T1^1^	58.123**	27	0.997	0.996	0.046	0.030–0.062
Gambling disorder LGC	0.308	1	1.000	1.000	0.000	0.000–0.088
Gambling disorder LGC alternative model	0.207**	0	0.991	1.000	0.000	0.000–0.000
Problematic pornography use CFA, T1	21.797**	9	0.997	0.995	0.045	0.021–0.069
Problematic pornography use CFA, T2	14.839	9	0.996	0.994	0.048	0.000–0.091
Problematic pornography use CFA, T3	15.240	9	0.995	0.992	0.062	0.000–114
Problematic pornography use LGC	1.145	1	0.999	0.997	0.014	0.000–0.098
Problematic pornography use LGC alternative model	0.148**	0	0.999	1.000	0.000	0.000–0.000
Compulsive sexual behavior disorder CFA, T1	661.599**	147	0.947	0.938	0.065	0.060–0.070
Compulsive sexual behavior disorder CFA, T2	389.494**	147	0.960	0.954	0.063	0.056–0.071
Compulsive sexual behavior disorder CFA, T3	302.724**	147	0.953	0.946	0.067	0.056–0.078
Compulsive sexual behavior disorder LGC	0.359	1	1.000	1.000	0.000	0.000–0.730
Compulsive sexual behavior disorder LGC alternative model	9.297**	0	0.958	1.000	0.000	0.000–0.000

*χ^2^, robust Chi-square test for exact of fit; df, degrees of freedom; CFI, comparative fit index; TLI, Tucker-Lewis index; RMSEA, root mean square error of approximation; 90% CI, confidence interval of the RMSEA; CFA, confirmatory factor analysis; LGC, latent growth-curve model; alternative model, the variance of the slope factor left infixed at the second data collection time-point.*

The three values in each box are the correlations at the three data collections (i.e., T1/T2/T3). ^1^In case of gambling disorder, the latent growth curve models were computed using the measured scale points, as the confirmatory factor analyses did not converge on T2 and T3 data collection waves. *p < 0.05; **p < 0.01.

**TABLE 4:** Growth curve models.

	*M*	SE	*p*-Value	Variance	SE	*p*-Value	*r*	*p*-Value
Problematic social media use intercept	0.060	0.030	0.044	0.665	0.055	<0.001		
Problematic social media use slope	–0.006	0.005	0.249	0.010	0.000	<0.001		
Standardized intercept-slope covariance							−0.046	0.000
Internet gaming disorder intercept	0.108	0.020	0.000	0.255	0.078	0.001		
Internet gaming disorder slope	–0.004	0.004	0.392	0.005	0.003	0.060		
Standardized intercept-slope covariance							−0.025	0.029
Gambling disorder intercept	9.294	0.074	0.000	2.577	0.937	0.006		
Gambling disorder slope	–0.015	0.009	0.088	0.001	0.000	<0.001		
Standardized intercept-slope covariance							−0.155	0.007
Problematic pornography use intercept	0.130	0.048	0.007	1.172	0.200	<0.001		
Problematic pornography use slope	–0.012	0.009	–0.182	0.014	0.004	0.002		
Standardized intercept-slope covariance							−0.052	0.100
Compulsive sexual behavior disorder intercept	0.105	0.031	0.001	0.412	0.058	<0.001		
Compulsive sexual behavior disorder slope	0.016	0.006	0.005	0.004	0.001	<0.001		
Standardized intercept-slope covariance							−0.011	0.155

*M, mean; SE, standard error.*

In the case of problematic social media use, the intercept showed significant and moderate variance, indicating that the sample varied in their initial levels of problematic social media use. The model showed no significant change over time, since the mean of the slope factor was not significant. Although the variance of the slope factor was significant, the variance itself remained negligible (see Table 4).

Based on the intercept factor, there was significant but low variability in the initial scores of the sample regarding Internet gaming disorder. However, neither the mean, nor the variance of the slope factor was significant. Therefore, there was no significant change over time regarding Internet gaming disorder.

In the case of gambling disorder, the LGCMs were calculated based on the assessed scale point of the PGSI, as confirmatory analyses did not converge on T2 and T3 time-points, not allowing the obtaining of latent factor scores. There was a high variability in the initial scores, suggesting that participants’ initial levels of gambling disorder at T1 varied significantly. The mean of the slope factor was not significant, indicating no change over time regarding gambling disorder.

Problematic pornography use’s linear trend was also calculated. The intercept was significant, and the variance of it was high, suggesting that the sample varied greatly in the initial scores of problematic pornography use. Although the variance of the slope was significant, it varied very little, and the slope was not significant, indicating no change over time in problematic pornography use.

Regarding compulsive sexual behavior disorder, the intercept showed moderate variability of the initial scores at T1. Although both the mean and the variance of the slope was significant, the small variance indicated negligible increases over time.

In sum, all LGCMs followed a linear trend and showed that the sample varied in their initial scores. However, no significant changes were observed over time in any of the examined problematic or addictive behaviors, except for compulsive behavior disorder, which demonstrated a small but significant increase.

## Discussion

To address concerns about the potential impact of the COVID-19 outbreak on potentially addictive behaviors, the aim of the present study was to assess longitudinal changes in addictive and problematic behaviors (i.e., problematic social media use, Internet gaming disorder, gambling disorder, problematic pornography use, and compulsive sexual behavior disorder) over time during the COVID-19 pandemic. Data collection took place over the course of the first and second wave of the pandemic in Hungary, starting at the beginning of the outbreak, when most of the restrictions came into force (May 2020), during the summer, when the situation was somewhat more relaxed (June–August 2020), and lastly, in the middle of the second wave of the pandemic, when even more strict rules were applied (January 2020). Results suggested no significant changes over the course of the 10 months of data collection in any of the studied problematic behaviors. These findings indicate that those who had no problem with these addictive behaviors at the first data collection, might have not developed a problem over time, and those who had problems with any of the behaviors, might have not experienced a significant increase in their symptoms. However, these finding**s** are based on data collected during the first wave of the pandemic and does not provide us about pre-pandemic levels of problematic behaviors. It is also important to note that in case of every problematic behavior, significant variability was observed at the initial scores and the changes over time as well. Meaning that the sample not only varied in their initial level of problematic behaviors, but that some participants might have experienced greater changes (either increase or decrease) in these behaviors over time than others. The present results are in line with previous, longitudinal findings about problematic pornography use during the pandemic (Grubbs et al., 2021; Bőthe et al., 2022) and contradict other longitudinal results about gaming disorder symptoms (Teng et al., 2021). However, the latter study assessed adolescents’ gaming behaviors, therefore, these findings might not be comparable to the present study or previous findings among adults (Grubbs et al., 2021).

Although several studies reported that participants might have experienced elevated self-perceived frequency of the examined behaviors based on retrospective assessment techniques (Håkansson, 2020; Gainsbury et al., 2021; Lugo et al., 2021; Sallie et al., 2021; Wardle et al., 2021), it seems that “hard data” and participants’ perception of their own behaviors do not completely overlap (Auer et al., 2020; Auer and Griffiths, 2021). Due to potential recall bias (Schmier and Halpern, 2004; Hipp et al., 2020), findings based on repeated measurements have stronger evidential values than studies using retrospective reports (i.e., asking participants to think back and report their use – frequency or symptom severity – at pre-pandemic times).

Since the first data collection wave was after the COVID-19 outbreak started (May 2020), it needs to be considered that the severity of these disorders might have already been elevated at the time of the first data collection point. Therefore, the findings might have assessed the already increased levels of symptoms throughout the pandemic. If this was the case, it would not be surprising that the analyses indicated no change over time, since the baseline measurement would have already deviated from the general levels of symptom-severity.

Moreover, it is important to note that for most of these potentially addictive behaviors with official diagnostic criteria, one key criterion is to experience symptoms for a prolonged period of time (e.g., at least for 6 months) to meet the diagnostic criteria (American Psychiatric Association, 2013; World Health Organization, 2019b). In the present study, participants were asked about their behaviors over the past 7 days. Therefore, even if some elevation had been traceable in the symptoms, it still would not have meant the trends met clinical thresholds.

Because of the high drop-out rates, it is also a possibility that participants were lost who had higher risk of developing problematic, out-of-control behaviors (for example, due to living situation, preexisting mental health issues, etc.). Previous studies examining longitudinal attrition analyses demonstrated that there is a chance of losing the most vulnerable participants at an early stage of data collection, when working with repeated measures (Štulhofer et al., 2021). However, attrition analysis did not show significant differences between participants who completed the follow-up surveys and those who dropped out during the follow-ups in the present study in their sociodemographic characteristics and initial levels of problematic behaviors. Nevertheless, it is possible that the COVID-19 pandemic and the related lockdowns impacted individuals’ mental health or addictive behaviors. It is possible that other addictive disorders (e.g., alcohol dependence or other substance use disorders), out of the scope of the present study, might have worsened and/or displaced the behaviors studied during these stressful times (Acuff et al., 2021; Xu et al., 2021). For example, alcohol use disorder was a prevalent health issue in Hungary before the pandemic (Horváth et al., 2019; World Health Organization, 2019a) and it might have increased during the COVID-19 pandemic.

### Limitations and Future Directions

Although this study examined longitudinal changes in five potentially addictive and problematic behaviors over a period of 10 months, some limitations (in addition to those already mentioned above) need to be noted. Beside the methodological advantages of the repeated measure design, the large drop-out rates resulted in small sample sizes by the final data collection wave. However, the use of the Full Information Maximum Likelihood method decreased the potential biases deriving from this limitation (Newman, 2003; Newman, 2014; Lang and Little, 2018). The lack of a pre-pandemic, baseline measurement is also a limitation of the present study. Although the linear slopes of the LGCMs were defined according to the months that passed after the COVID-19 outbreak in Hungary (T1 assessment was fixed to two instead of the standard zero), these adjustments cannot substitute for a baseline data collection. However, as the outbreak of a global pandemic was unpredictable, the research team were not able to plan for a baseline data collection before the pandemic. As in the case of studies operating with a large body of survey battery, the study was sparing with the number of scales and items used to minimize survey fatigue. Therefore, several theoretically driven addictive behaviors were not included in the present study (e.g., compulsive online shopping, exercise addiction, and work addiction). In the present study, a convenience sample of the general population was used, limiting the generalizability of the results to participants who have Internet access. Furthermore, exploring the co-occurrence among different problematic behaviors would have also been a relevant study aim. However, the present study did not have a sufficient sample size to conduct such a complex analysis.

Lastly, using self-report online scales to assess problematic and addictive behaviors and symptoms could potentially lead to biases (e.g., underreporting, or overreporting, and social desirability bias). While the examined disorders and symptoms did not worsen over time during the COVID-19 pandemic in the general population, it is possible that a smaller number of participants (i.e., who have been at risk by the beginning of the lockdowns already) would have indicated change over time. But since the present study focused on a general trend, the possible effect was not detectable. Therefore, future studies could focus on more specific samples instead of the general population, such as individuals with pre-existing mental health disorders (Bonny-Noach and Gold, 2021). Alternatively, person-centered approaches could offer a solution for this issue in the future (e.g., mixture models). Furthermore, including theoretically justified control variables (e.g., living situation and health-anxiety) could also result in more specific knowledge on the potential impacts of the pandemic.

## Conclusion

Contrary to initial concerns (Király et al., 2020; Mestre-Bach et al., 2020; Singh et al., 2020; Awan et al., 2021; Marchi et al., 2021), no substantial changes were detected over time in problematic and potentially addictive behaviors (i.e., problematic social media use, Internet gaming disorder, gambling disorder, problematic pornography use, and compulsive sexual behavior disorder) during the COVID-19 pandemic. The present study also addressed another concern regarding more frequent engagement in screen-based activities during the COVID-19 pandemic. The present results are in line with previous studies suggesting that more frequent engagement in a given activity might not be a sufficient indicator of problematic use (Grubbs et al., 2019; Bőthe et al., 2020c). In this sense, elevated frequency of use during the COVID-19 pandemic might not necessarily result in developing problematic and out-of-control behaviors.

## Data Availability Statement

The raw data supporting the conclusions of this article will be made available by the authors, without undue reservation.

## Ethics Statement

The studies involving human participants were reviewed and approved by the Eötvös Loránd University, Budapest, Hungary. The patients/participants provided their written informed consent to participate in this study. Written informed consent was obtained from the individual(s) for the publication of any potentially identifiable images or data included in this article.

## Author Contributions

MK, BB, and ZD: conception and design. MK and BB: analysis of data. MK, BB, MG, and ZD: interpretation of data. MK: drafting the manuscript. BB, MG, and ZD: revising the manuscript critically for important intellectual content. All authors contributed to the article and approved the submitted version.

## Conflict of Interest

Eötvös Loránd University (ELTE) receives funding from the Szerencsejáték Ltd. to maintain a telephone helpline service for problematic gambling. ZD has also been involved in research on responsible gambling funded by Szerencsejáték Ltd. and the Gambling Supervision Board and provided educational materials for the Szerencsejáték Ltd.’s responsible gambling program. The University of Gibraltar receives funding from the Gibraltar Gambling Care Foundation. However, these funding are not related to this study and the funding institution had no role in the study design or the collection, analysis, and interpretation of the data, writing the manuscript, or the decision to submit the article for publication. MG’s university currently receives research funding from Norsk Tipping (the gambling operator owned by the Norwegian Government). MG has also received funding for a number of research projects in the area of gambling education for young people, social responsibility in gambling and gambling treatment from Gamble Aware (formerly the Responsible Gambling Trust), a charitable body which funds its research program based on donations from the gambling industry. MG regularly undertakes consultancy for various gaming companies in the area of social responsibility in gambling. The remaining authors declare that the research was conducted in the absence of any commercial or financial relationships that could be construed as a potential conflict of interest.

## Publisher’s Note

All claims expressed in this article are solely those of the authors and do not necessarily represent those of their affiliated organizations, or those of the publisher, the editors and the reviewers. Any product that may be evaluated in this article, or claim that may be made by its manufacturer, is not guaranteed or endorsed by the publisher.

## Footnotes

1 Problematic social media use: Δχ^2^[1] = 2.85, *p* = 0.221; Internet gaming disorder: Δχ^2^[1] = −0.42, *p* = 0.110; gambling disorder: Δχ^2^[1] = 20.07, *p* = 0.585; problematic pornography use: Δχ^2^[1] = 0.94, *p* = 0.301; compulsive sexual behavior disorder (Δχ^2^[1] = 0.97, *p* = 0.325).
