# Supplementary material for: No Significant Changes in Addictive and Problematic Behaviors During the COVID-19 Pandemic and Related Lockdowns: A Three-Wave Longitudinal Study
Source: Front Psychol. 2022 Apr 13;13:837315. doi: 10.3389/fpsyg.2022.837315 (PMC9043320; doi:10.3389/fpsyg.2022.837315)
Supplement: Supplementary file 1 [file Data_Sheet_1.docx]

Appendix A. - Sociodemographic characteristics of the sample at the three data collection waves

|  |  | T1  (N = 1747) | T2  (N = 656) | T3  (N = 411) |
| --- | --- | --- | --- | --- |
| Gender | Men | 882 | 273 | 172 |
|  | Women | 846 | 358 | 234 |
|  | Gender diverse individuals | 17 | 9 | 5 |
| Age (in years) | M (SD) | 41.96 (12.52) | 42.89 (13.10) | 43.01 (13.43) |
|  | Range | 18 - 80 | 18-80 | 18-80 |
| Sexual orientation | Heterosexual | 1206 | 520 | 346 |
|  | Bisexual | 39 | 18 | 12 |
|  | Homosexual | 35 | 17 | 7 |
|  | Asexual | 22 | 12 | 6 |
|  | Unsure | 6 | 4 | 3 |
|  | Other | 12 | 6 | 6 |
| Education | Primary school degree or less | 7 | 7 | 2 |
|  | Vocational degree | 100 | 39 | 20 |
|  | High school degree | 329 | 106 | 63 |
|  | Higher education degree (e.g., bachelors, masters or doctorate) | 1306 | 493 | 326 |
| Relationship status | Single | 482 | 163 | 105 |
|  | In a relationship | 473 | 174 | 112 |
|  | Married/Common- law partner* | 590 | 238 | 147 |
|  | Divorced | 28 | 11 | 11 |
|  | Widowed | 93 | 45 | 31 |
| Studying currently |  | 260 | 97 | 66 |
| Working currently |  | 1479 | 540 | 345 |
| Residence | Large city (over 100,000 citizens) | 1159 | 436 | 287 |
|  | Large city (100,000 person – 999,999 citizens) | 227 | 82 | 51 |
|  | City (below 100,000 citizens) | 325 | 118 | 71 |
|  | Village | 149 | 71 | 45 |
|  | Other | 31 | 6 | 3 |
| Socio-economic status | Among the best | 24 | 10 | 5 |
|  | Much better than average | 436 | 158 | 103 |
|  | Little bit better than average | 632 | 248 | 168 |
|  | Average | 458 | 172 | 102 |
|  | Little bit worse than average | 86 | 34 | 25 |
|  | Much worse than average | 15 | 4 | 2 |
|  | Among the worst | 3 | 1 | 0 |

*Note*. M = mean; SD = standard deviation.
