# Supplementary material for: No Significant Changes in Addictive and Problematic Behaviors During the COVID-19 Pandemic and Related Lockdowns: A Three-Wave Longitudinal Study
Source: Front Psychol. 2022 Apr 13;13:837315. doi: 10.3389/fpsyg.2022.837315 (PMC9043320; doi:10.3389/fpsyg.2022.837315)
Supplement: Supplementary file 2 [file Data_Sheet_2.docx]

Appendix B

Comparison of participants who completed vs. who dropped-out at the second, vs. who dropped-out at third data collection wave in their demographic characteristics at baseline (T1) measurements

| Variables | (1)  Completed the survey  (N = 417)  *n* (%) | (2)  Dropped-out at T2  (N = 608)  *n* (%) | (3)  Dropped-out at T3  (N = 487)  *n* (%) | *χ*^2^ test | *p* | Cramer’s *V* |
| --- | --- | --- | --- | --- | --- | --- |
| Gender |  |  |  | 26.78 | < .001* | .09 |
| Men | 171^2^ | 608^1,3^ | 102^2^ |  |  |  |
| Women | 233^2^ | 487^1,3^ | 125^2^ |  |  |  |
| Other | 5 | 8 | 4 |  |  |  |
| Sexual orientation |  |  |  | 5.63 | .060 | .065 |
| Heterosexual | 345 | 685 | 175 |  |  |  |
| Sexually diverse | 31 | 57 | 26 |  |  |  |
| Relationship status |  |  |  | 2.40 | .301 | .038 |
| Single/Divorced/Widowed | 147 | 384 | 72 |  |  |  |
| In a relationship | 257 | 649 | 155 |  |  |  |
| Variables | *M (SD); Median* | *M (SD); Median* | *M (SD); Median* | Kruskal-Wallis test | *p* | *η²* |
| Age in years | 42.97 (13.40); 42 | 41.40 (12.13); 41 | 42.76 (12.57); 42 | 3.91 | .142 | 0.003 |
| Education^a^ | 8.00 (1.67); 9 | 7.84 (1.72); 8 | 7.85 (1.77); 9 | 3.93 | .140 | 0.001 |
| Residence^b^ | 1.84 (1.12); 1 | 1.87 (1.12); 1 | 1.95 (1.15); 1 | 1.48 | .476 | <0.001 |
| Socio-economic status^c^ | 4.89 (0.93); 5 | 4.90 (0.99); 5 | 4.90 (1.03); 5 | 0.07 | .965 | <0.001 |
| Problematic social media use at T1  (range: 6 - 30) | 10.02 (4.15)^2^; 9 | 9.46 (3.92)^1^; 8 | 9.37 (3.83); 8 | 6.65 | .036 | 0.004 |
| Online gaming disorder at T1 (range: 0-9) | 0.41 (1.01); 0 | 0.34 (0.96); 0 | 0.33 (0.82); 0 | 0.98 | .614 | <0.001 |
| Gambling disorder at T1 (range: 9- 36) | 9.10 (0.57); 9 | 9.27 (1.47); 9 | 9.23 (0.97); 9 | 2.40 | .302 | 0.004 |
| Problematic pornography use at T1  (range: 6 - 49) | 9.91 (5.55); 7 | 10.22 (5.96); 7 | 9.33 (5.23); 7 | 2.51 | .286 | 0.003 |
| Compulsive sexual behavior disorder at T1 (range: 19 - 76) | 24.08 (5.97); 22 | 24.40 (7.07); 22 | 24.28 (6.59); 22 | 0.06 | .971 | <0.001 |

*Note. M* = mean; *SD* = standard deviation; T1 = Time 1 data collection; T2 = Time 2 data collection; T3 = Time 3 data collection. Superscript numbers (1, 2, 3) indicate significant (*p* < .05) difference between the given group and the indexed group within the same variable.

^a^1 = less than 8 years of elementary school, 2 = 8 years of elementary school, 3 = trade school; vocational training without high school diploma, 4 = vocational secondary school, 5 = high school diploma, 6 = intermediate level technical institute, 7 = higher forms vocational training; higher forms technical institute (not college), 8 = Bachelor’s degree / BA / BSC, 9 = Master’s degree / MA / MSC, 10 = postgraduate training, doctoral school (PhD, DLA);

^b^1 = metropolis (over 1 million citizens), 2 = large city (100,000 person – 999,999 citizens), 3 = city (below 100,000 citizens), 4 = village, 5 = other;

^c^1 = my life circumstances are among the worsts, 2 = my life circumstances are much worse than average, 3 = my life circumstances are worse than average, 4 = my life circumstances are average, 5 = my life circumstances are better than average, 6 = my life circumstances are much better than average, 7 = my life circumstances are among the bests.
